# pH-Responsive Apalutamide-Loaded ZIF‑8 Nanoparticles for Zinc-Enhanced Dual Cytotoxicity in Prostate Cancer

**DOI:** 10.1021/acsomega.6c01591

**Published:** 2026-08-17

**Authors:** Derya Mete, Gülşah Şanlı-Mohamed

**Affiliations:** 52972 İzmir Institute of Technology, Department of Chemistry, İzmir 35430, Turkey

## Abstract

Apalutamide (APA) is a recently FDA-approved androgen receptor inhibitor for the treatment of nonmetastatic castration-resistant prostate cancer. Herein, we report a pH-responsive, dual-acting nanocarrier system based on zeolitic imidazolate framework-8 (ZIF-8) to enhance the therapeutic efficacy of APA. APA was successfully encapsulated into ZIF-8 nanoparticles with an encapsulation efficiency of 69.4% and a drug loading capacity of 47.8% (APA@ZIF-8). The structural and physicochemical properties of ZIF-8 and APA@ZIF-8 were comprehensively characterized using FTIR, SEM, STEM, DLS, EDX, PXRD, TGA, and BET analyses. Drug release studies demonstrated a pronounced pH-dependent behavior, with a faster release at pH 5.0 (93% over 120 h) compared to pH 7.4 (62% over 120 h), indicating suitability for the acidic tumor microenvironment. Biocompatibility assessments, including hemolysis and serum protein binding assays, confirmed the hemocompatible nature of both ZIF-8 and APA@ZIF-8 nanoparticles. The biological effects of APA, ZIF-8, and APA@ZIF-8 were evaluated through cell viability, cell cycle progression, reactive oxygen species generation, apoptosis, and androgen receptor transcriptional activity in prostate cancer and healthy cell lines. The results reveal that the pH-triggered biodegradation of ZIF-8 leads to zinc ion release, which, together with APA, contributes to enhanced cytotoxic effects in prostate cancer cells, highlighting the potential of APA@ZIF-8 as a zinc-enhanced dual cytotoxic nanotherapeutic platform.

## Graphical abstract

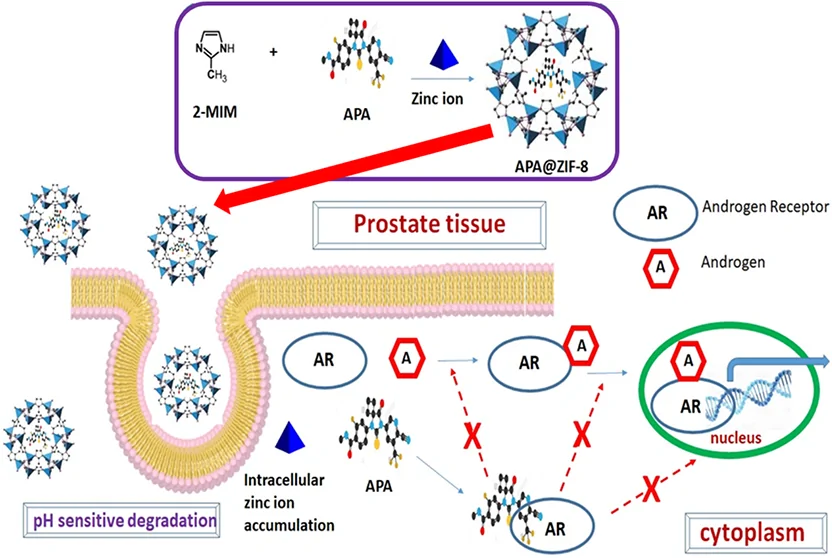

## 1 Introduction

Prostate cancer is the most common internal malignancy in men and the second leading cause of cancer-related mortality worldwide.^1,2^ The disease is often asymptomatic in its early stages and may remain undetected until it becomes advanced. Early diagnosis is generally based on elevated prostate-specific antigen (PSA) levels in blood together with digital rectal examination. Androgen-deprivation therapy is the standard initial treatment strategy and may be achieved by bilateral orchiectomy or the administration of gonadotropin-releasing hormone analogues. Although this approach is initially effective, most patients ultimately progress to castration-resistant prostate cancer (CRPC).^3^ Recent epidemiological and clinical data further emphasize the increasing global burden of prostate cancer and the need for improved therapeutic strategies.^2^

Apalutamide (also known as ARN-509, Erleada, or JNJ-56021927) is a next-generation androgen receptor (AR) inhibitor approved by the U.S. Food and Drug Administration (FDA) in 2018 for the treatment of nonmetastatic castration-resistant prostate cancer (CRPC).^4^ Chemically, apalutamide is 4-[7-[6-cyano-5-(trifluoromethyl)­pyridin-3-yl]-8-oxo-6-sulfanylidene-5,7-diazaspiro[3.4]­octan-5-yl]-2-fluoro-*N*-methylbenzamide. It exerts its therapeutic effect by binding to the ligand-binding domain of AR, thereby inhibiting AR nuclear translocation, DNA binding, and subsequent AR-mediated transcriptional signaling.^5,6^ Owing to this mechanism, apalutamide has become an important therapeutic agent for the management of nonmetastatic CRPC.^4,5,7^ Recent advances in androgen receptor signaling inhibitors have further highlighted the clinical importance of AR-targeted therapies in improving survival outcomes in advanced prostate cancer.^7^ Despite its clinical efficacy, the therapeutic potential of apalutamide may be limited by insufficient tumor-selective delivery, systemic exposure, and suboptimal intracellular accumulation. These limitations highlight the need for advanced delivery strategies to further improve its therapeutic performance.

In recent years, nanotechnology-based drug delivery systems have attracted considerable attention because they can improve drug stability, enable controlled release, enhance pharmacokinetic behavior, and promote tumor-targeted delivery.^8^ Such systems may increase therapeutic efficacy while reducing systemic adverse effects. Among these systems, metal–organic frameworks (MOFs) have emerged as highly promising materials owing to their high surface area, tunable pore structures, structural versatility, and high loading capacity.^9–11^ MOFs are crystalline porous materials formed by the coordination of metal ions with multidentate organic ligands, enabling the design of well-defined architectures with adjustable physicochemical properties.^10,12^ These features, together with their functional diversity and biocompatibility, have led to extensive investigation of MOFs in applications such as catalysis, gas storage, separation, and drug delivery^9,11,13^ Recent advances have further demonstrated that MOF-based nanoplatforms can be engineered as multifunctional systems responsive to tumor microenvironment stimuli, thereby enabling synergistic therapeutic effects that combine drug delivery with the intrinsic activity of the carrier material.^12^

Zeolitic imidazolate framework-8 (ZIF-8), composed of zinc ions coordinated with 2-methylimidazole, is one of the most extensively studied MOFs for biomedical applications. Owing to its high loading capacity, biocompatibility, and pH-responsive degradation behavior, ZIF-8 has emerged as an attractive platform for drug delivery and controlled release, particularly in acidic tumor microenvironments.^14^ Beyond its role as a passive carrier, ZIF-8 can also contribute to therapeutic efficacy through the release of Zn^2+^ ions upon degradation. These ions are known to disrupt mitochondrial function, induce oxidative stress, and promote apoptosis in cancer cells, thereby providing an additional therapeutic advantage. ZIF-8 has been used to encapsulate a wide range of molecules, including methyl orange, methylene blue, caffeine, cytochrome c, curcumin, 3-methyladenine (3-MA), and insulin,^15–20^ and has been widely explored as a drug delivery system for therapeutic agents such as doxorubicin, 5-fluorouracil, camptothecin, 6-mercaptopurine, paclitaxel, and doxepin.^21–26^ Recent studies have further demonstrated the effectiveness of ZIF-8-based nanoplatforms in cancer therapy and stimuli-responsive drug delivery systems.^24,27^

Zinc homeostasis is essential for normal prostate physiology, and healthy prostate tissue accumulates substantially higher levels of zinc than most other soft tissues. In prostate cancer, however, this homeostatic balance is disrupted, and malignant cells exhibit a reduced capacity to accumulate zinc, particularly in castration-resistant disease.^28,29^ This decline in intracellular zinc levels is associated with the downregulation of zinc uptake transporters such as ZIP-1.^9^ Zinc is involved in multiple cellular processes relevant to cancer biology, including p53-related signaling, mitochondrial function, citrate metabolism, and oxidative stress pathways.^30,31^ Restoration of intracellular zinc levels has been shown to induce cytotoxic effects in prostate cancer cells through mechanisms involving mitochondrial dysfunction, reactive oxygen species generation, and apoptosis.^31,32^ Therefore, zinc-mediated cytotoxicity represents a biologically relevant strategy that may complement conventional prostate cancer therapies.

Despite the individual therapeutic potential of apalutamide and zinc-based cytotoxic mechanisms, their integration within a single nanoplatform remains largely unexplored. To the best of our knowledge, the encapsulation of apalutamide within a ZIF-8-based nanocarrier to simultaneously exploit controlled drug delivery and zinc-mediated synergistic effects has not yet been reported.

In this study, we report the design and development of a pH-responsive ZIF-8-based nanocarrier (APA@ZIF-8) for enhanced prostate cancer therapy. By integrating controlled apalutamide release with Zn^2+^-mediated cytotoxicity, the proposed system aims to achieve a synergistic therapeutic effect. Apalutamide was encapsulated within the ZIF-8 framework, a zinc-based metal–organic framework composed of zinc ions and 2-methylimidazole, enabling both drug delivery and ion-mediated therapeutic activity. The nanocarrier was systematically characterized, and its biological performance was evaluated through cell viability, cell cycle progression, reactive oxygen species generation, apoptosis, and androgen receptor transcriptional activity assays. This dual-function nanotherapeutic platform combines hormone therapy with metal ion-mediated mechanisms, providing a multifunctional, biocompatible, and biodegradable strategy for the treatment of advanced prostate cancer.

## 2 Materials and Methods

### 2.1 Synthesis of ZIF-8 and APA@ZIF-8 Nanoparticles

ZIF-8 nanoparticles were synthesized via a one-pot method with minor modifications based on previously reported procedures.^21,33^ A molar ratio of Zn^2+^:2-methylimidazole (2-MeIm):H_2_O = 1:70:1238 was used for the synthesis. Specifically, 585 mg of zinc nitrate hexahydrate [Zn­(NO_3_)_2_·6H_2_O] was dissolved in 4 mL of deionized water to prepare the metal precursor solution. Separately, 11.35 g of 2-methylimidazole (2-MeIm) was dissolved in 40 mL of deionized water, followed by the addition of 6 mL of dimethyl sulfoxide (DMSO) to improve solubility and dispersion.

The zinc nitrate solution was then added to the ligand solution under stirring at room temperature (∼25 °C), resulting in the immediate formation of a milky white suspension. The mixture was stirred for 5 min and subsequently centrifuged at 14,000 rpm for 15 min. The obtained precipitate was washed three times with ethanol to remove unreacted species and then dried at 65 °C overnight, yielding ZIF-8 nanoparticles as a white powder.

For the synthesis of APA@ZIF-8, 60 mg of apalutamide was first dissolved in 6 mL of DMSO and added to the 2-MeIm solution prior to the addition of the zinc precursor. The subsequent steps were carried out following the same procedure as described for ZIF-8. The resulting product was collected, washed, and dried under identical conditions to obtain APA-loaded ZIF-8 nanoparticles.

To ensure reproducibility and consistency of the synthesis process, all experiments were performed under identical conditions.

#### 2.1.1 Yield, Drug Loading, and Encapsulation Efficiency

The synthesis yield of the nanoparticles was determined gravimetrically after washing and drying steps. Drug loading capacity and encapsulation efficiency were evaluated using inductively coupled plasma–optical emission spectrometry (ICP-OES) (Agilent, USA). Prior to analysis, the samples were digested in a 5% aqueous HNO_3_ solution to ensure complete decomposition of the nanoparticles.

The encapsulation efficiency (EE%) and drug loading (DL%) were calculated using the following equations:

$$\mathrm{Encapsulation Efficiency}\;\%=\frac{\mathrm{Amount of LAP encapsulated}}{\mathrm{Amount of the prepared solution of LAP}}\times 100$$

$$\mathrm{Drug Loading}\;\%=\frac{\mathrm{Amount of LAP encapsulated}}{\mathrm{Total amount of nanoparticle}}\times 100$$

### 2.2 ZIF-8 And APA@ZIF-8 Nanoparticles Characterization

#### 2.2.1 Structural Analysis

Various analytical techniques were employed to characterize the synthesized nanoparticles in terms of their size, size distribution, surface charge, morphology, elemental composition, crystallinity, and functional groups. The hydrodynamic particle size and zeta potential of ZIF-8 and APA@ZIF-8 nanoparticles were measured using dynamic light scattering (DLS) with a Zetasizer Nano Z (Malvern Panalytical, UK). The morphology and structural features of the nanoparticles were examined using scanning electron microscopy (SEM) and scanning transmission electron microscopy (STEM). The samples were imaged using an FEI QUANTA 250 FEG instrument (USA) operated at an accelerating voltage of 5 kV, providing detailed information on particle size, surface morphology, and structural integrity. The elemental composition of the nanoparticles was analyzed by energy-dispersive X-ray spectroscopy (EDX). The crystalline structure of ZIF-8 and APA@ZIF-8 nanoparticles was investigated using powder X-ray diffraction (PXRD) (Philips X’Pert Pro diffractometer, Royal Philips Electronics, Amsterdam, The Netherlands) with Cu Kα radiation (λ = 1.541 Å) operated at 40 kV and 25 mA. The functional groups of the nanoparticles were identified using Fourier transform infrared spectroscopy (FTIR) (PerkinElmer, USA). Thermal stability of the nanoparticles was evaluated using thermogravimetric analysis (TGA). These characterization techniques collectively provided comprehensive insight into the physicochemical properties of the synthesized nanoparticles.

#### 2.2.2 Zn^2+^ Concentration Determination

ZIF-8 and APA@ZIF-8 nanoparticles were dispersed in three different buffer solutions: phosphate–citrate buffer (pH 5.0 and 6.0) and phosphate-buffered saline (PBS, pH 7.4). The suspensions were stirred at 150 rpm for 5 days at room temperature, followed by centrifugation to separate the solid particles. The supernatant was carefully collected and subjected to acid digestion prior to analysis. For this purpose, the samples were diluted with hydrochloric acid (HCl) and deionized (DI) water at appropriate ratios (1:10 or 1:50, v/v). The concentration of released Zn^2+^ ions was then determined using flame atomic absorption spectroscopy (FAAS) at a wavelength of 213.9 nm.

#### 2.2.3 *In Vitro* Release of APA from APA@ZIF-8

The pH-dependent in vitro release of apalutamide from APA@ZIF-8 nanoparticles was evaluated in PBS at pH 7.4 and 5.0 at 37 °C. Briefly, 3 mg of APA@ZIF-8 was suspended in 1 mL of buffer and incubated under continuous shaking. At selected time points over 120 h, the samples were centrifuged, and the supernatant was withdrawn for analysis. An equal volume of fresh buffer was added after each sampling step to maintain the release medium volume. APA concentration in the collected supernatants was determined by UV–vis spectroscopy (Shimadzu UV-2550, Japan) at 270 nm, which corresponds to the maximum absorbance wavelength of APA. The cumulative release of APA was calculated based on the measured drug concentration at each time point. The cumulative release % of APA was calculated according to the following equation

$$\mathrm{Cumulative Drug Release}\;\%=\sum_{i=1}^{n}M_{i}/M_{0}\times 100$$

where *M*_i_ is the amount of APA released from APA@ZIF-8 at time *i* and *M*_0_ is the total amount of loaded drug in APA@ZIF-8.

#### 2.2.4 Biocompatibility Studies

Biocompatibility of the synthesized nanoparticles was evaluated through serum protein binding and hemolysis assays.

##### 2.2.4.1 Serum Protein Binding Assay

Protein binding of the nanoparticles was assessed using fetal bovine serum (FBS) and quantified by the Bradford protein assay.^34^ Nanoparticle–protein mixtures were prepared at different volumetric ratios (10:90, 20:80, 40:60, and 60:40, v/v) to a final volume of 1000 μL. The samples were incubated at 37 °C under gentle shaking (150 rpm) for 2 h. Following incubation, the samples were centrifuged at 13,500 rpm for 15 min to separate nanoparticle-bound proteins from the supernatant. The concentration of unbound protein in the supernatant was determined spectrophotometrically at 595 nm using a bovine serum albumin (BSA) calibration curve. The amount of protein bound to the nanoparticles was calculated by subtracting the unbound protein from the initial protein concentration. The protein binding percentage was calculated according to the following equation

$$\mathrm{Protein Binding}\;\%=\frac{\mathrm{Concn}\;\left(\mathrm{initial protein}\right)-\mathrm{Concn}\;\left(\mathrm{unbound protein}\right)}{\mathrm{Concn}\;\left(\mathrm{initial protein}\right)}\times 100$$

##### 2.2.4.2 Hemocompatibility (Hemolysis) Assay

Hemocompatibility of the nanoparticles was evaluated using an in vitro hemolysis assay as previously described.^33,35^ Human erythrocytes were isolated by centrifugation and washed with phosphate-buffered saline (PBS, pH 7.4). The erythrocytes were diluted to obtain a 2% suspension in PBS. Nanoparticle suspensions at concentrations of 2, 10, and 20 μg/mL were mixed with erythrocytes at a 1:1 (v/v) ratio and incubated at 37 °C under gentle shaking (100 rpm) for 4 h. PBS and 1% Triton X-100 were used as negative (0% hemolysis) and positive (100% hemolysis) controls, respectively. After incubation, the samples were centrifuged at 4100 rpm for 10 min, and the supernatants were collected. The released hemoglobin was quantified by measuring the absorbance at 540 nm using a UV–vis spectrophotometer. The hemolysis percentage was calculated using the following equation

$$\mathrm{Hemolysis}\;\%=\frac{\mathrm{Absorbance}\;\left(\mathrm{sample}\right)-\mathrm{Absorbance}\;\left(\mathrm{negative control}\right)}{\mathrm{Absorbance}\;\left(\mathrm{positive control}\right)}\times 100$$

### 2.3 Cell Culture Studies

Prostate cancer cell lines (LNCaP, PC3, and 104R2) were obtained from the Bioengineering Department of Ege University (Türkiye). LNCaP cells are androgen-sensitive, whereas PC3 and 104R2 cells are androgen-independent. LNCaP cells were cultured in RPMI 1640 medium, while PC3 and 104R2 cells were maintained in DMEM/F-12 medium. All media were supplemented with 10% fetal bovine serum (FBS), 1% penicillin–streptomycin, and 1% l-glutamine. Cells were incubated at 37 °C in a humidified atmosphere containing 5% CO_2_ and were passaged upon reaching approximately 80% confluency.

#### 2.3.1 Cell Viability and Cytotoxicity Analysis

Cell viability was evaluated using the WST-1 assay.^36^ LNCaP, PC3, and 104R2 cells were seeded in 96-well plates at a density of 5 × 10^3^ cells per well and incubated for 24 h at 37 °C in a humidified atmosphere containing 5% CO_2_. After incubation, the cells were treated with varying concentrations of ZIF-8 and APA@ZIF-8 nanoparticles. Prior to treatment, the nanoparticle suspensions were sterilized by UV irradiation. The cells were then incubated for 24, 48, and 72 h. At each time point, 10 μL of WST-1 reagent was added to each well and incubated for an additional 2 h. The absorbance was measured at 440 nm using a microplate reader. Cell viability was expressed as a percentage relative to untreated control cells. All experiments were performed in triplicate.

#### 2.3.2 Apoptosis Analysis

Apoptosis was evaluated using an Annexin V-FITC/propidium iodide (PI) assay (BioVision, USA). LNCaP and PC3 cells were seeded in 6-well plates at a density of 5 × 10^5^ cells per well and incubated for 24 h at 37 °C in a humidified atmosphere containing 5% CO_2_. Following incubation, the cells were treated with ZIF-8 and APA@ZIF-8 nanoparticles at concentrations of 5, 10, and 20 μg/mL for 24 h. After treatment, the cells were harvested, washed with phosphate-buffered saline (PBS), and stained with Annexin V-FITC and PI according to the manufacturer’s instructions. The stained cells were analyzed using a flow cytometer (BD FACSCanto, USA). Data were processed to determine the percentages of viable, early apoptotic, late apoptotic, and necrotic cells. All experiments were performed in triplicate.

#### 2.3.3 Cell Cycle Analysis

Cell cycle distribution was analyzed using propidium iodide (PI) staining (BioVision, USA). LNCaP and PC3 cells were seeded in 6-well plates at a density of 5 × 10^5^ cells per well and incubated for 24 h at 37 °C in a humidified atmosphere containing 5% CO_2_. The cells were then treated with ZIF-8 and APA@ZIF-8 nanoparticles at concentrations of 5, 10, and 20 μg/mL for 24 h. After treatment, the cells were harvested and fixed in cold 99.8% ethanol, followed by incubation at −20 °C for at least 24 h. The fixed cells were washed and resuspended in phosphate-buffered saline containing 0.1% Triton X-100 and treated with RNase A and propidium iodide. DNA content was analyzed by flow cytometry (BD FACSCanto, USA), and cell cycle distribution (G0/G1, S, and G2/M phases) was determined using ModFit LT software. A minimum of 10,000 events were recorded for each sample. All experiments were performed in triplicate.

#### 2.3.4 Reactive Oxygen Species (ROS) Detection Analysis

Intracellular reactive oxygen species levels were measured using a Reactive Oxygen Species Detection Assay Kit (BioVision, USA) according to the manufacturer’s protocol. LNCaP and PC3 cells were seeded in appropriate culture plates and incubated for 24 h at 37 °C in a humidified atmosphere containing 5% CO_2_. Following incubation, the cells were treated with ZIF-8 and APA@ZIF-8 nanoparticles for 24 h. After treatment, the cells were washed with phosphate-buffered saline (PBS) and incubated with the ROS-sensitive fluorescent probe (DCFH-DA) at 37 °C for 30 min in the dark. After incubation, excess dye was removed by washing with PBS. Fluorescence intensity was measured using a microplate reader at excitation and emission wavelengths of 495 and 529 nm, respectively. The relative ROS levels were expressed as normalized fluorescence intensity compared to untreated control cells. All experiments were performed in triplicate..

#### 2.3.5 Reporter Activity Analysis

Androgen receptor (AR) transcriptional activity was evaluated using a dual-luciferase reporter assay. LNCaP cells were seeded in 48-well plates at a density of 1 × 10^5^ cells per well and incubated for 24 h at 37 °C in a humidified atmosphere containing 5% CO_2_. The cells were transiently transfected with androgen-responsive reporter plasmids using Fugene transfection reagent (Promega) according to the manufacturer’s protocol. After 24 h of transfection, the cells were treated with APA, ZIF-8, APA@ZIF-8, and the synthetic androgen R1881 for an additional 24 h. Following treatment, the cells were washed with phosphate-buffered saline (PBS) and lysed using passive lysis buffer. Luciferase activity was measured using a dual-luciferase reporter assay kit (Promega, Mannheim, Germany) according to the manufacturer’s instructions. Firefly luciferase activity was normalized to Renilla luciferase activity to account for transfection efficiency. All experiments were performed in triplicate.

### 2.4 Data Presentation and Statistical Analysis

All experiments were performed in triplicate, and the results are expressed as mean ± standard deviation (SD). Statistical analysis was carried out using two-way analysis of variance (ANOVA) followed by an appropriate multiple-comparison post hoc test. A value of *p* < 0.05 was considered statistically significant.

## 3 Results and Discussion

ZIF-8 and APA@ZIF-8 nanoparticles were successfully synthesized using a one-pot method. A key feature of ZIF-8 is its pH-responsive stability, which makes it a suitable candidate for controlled drug release applications.

To evaluate the structural stability of ZIF-8 under different physiological conditions, the nanoparticles were incubated at pH 7.4, 6.0, and 5.0 for 5 days at room temperature. The release of Zn^2+^ ions into the supernatant was quantified using atomic absorption spectroscopy (AAS). As shown in Figure 1A, Zn^2+^ release increased with decreasing pH, indicating enhanced degradation of the ZIF-8 framework under acidic conditions. This behavior can be attributed to the weakening of coordination bonds between zinc ions and imidazolate linkers at lower pH levels. At a concentration of 30 μg mL^–1^, the amount of released Zn^2+^ ranged between 3–5 μg mL^–1^. These values are comparable to physiological zinc levels reported in human blood (3–6.4 μg mL^–1^),^37^ suggesting that the released zinc concentrations remain within a biologically relevant range. Overall, the results confirm the pH-sensitive degradation behavior of ZIF-8, which is advantageous for tumor-targeted drug delivery in acidic microenvironments.

**Figure 1:**
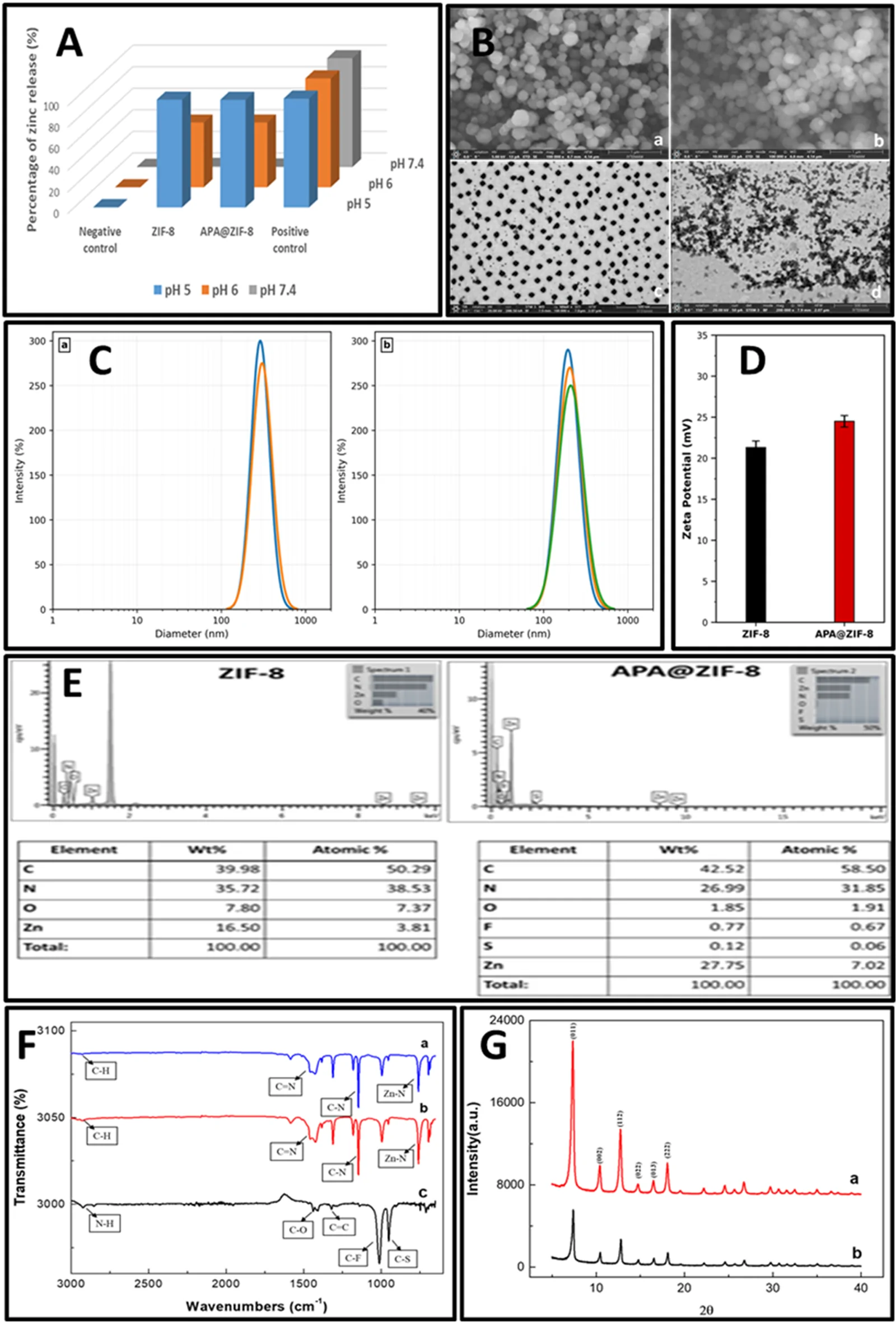
(A) Zinc release from ZIF-8 and APA@ZIF-8 at pH 7.4, 6.0, and 5.0. (B) SEM and STEM micrographs of (a, c) ZIF-8 and (b, d) APA@ZIF-8, respectively. (C) DLS measurements of (a) ZIF-8 and (b) APA@ZIF-8 (D) Zeta potentials of ZIF-8 and APA@ZIF-8 (E) EDX analysis of ZIF-8 and APA@ZIF-8. (F) PXRD patterns of (a) ZIF-8 and (b) APA@ZIF-8. (G) FTIR spectra for (a) ZIF-8 (b) APA@ZIF-8 and (c) APA.

The successful incorporation of apalutamide into the ZIF-8 framework was confirmed by ICP-OES analysis. The APA@ZIF-8 nanoparticles exhibited a drug loading capacity of 47.8% and an encapsulation efficiency of 69.4%, demonstrating the effectiveness of the one-pot synthesis strategy. The loading behavior is governed by the interactions between the drug molecules, solvent environment, and the porous internal structure of ZIF-8, which collectively determine the adsorption efficiency. For ICP-OES measurements, the nanoparticles were digested in 5% HNO_3_ to quantify the Zn content, which was subsequently used to calculate the ZIF-8 yield. The corresponding drug loading values are summarized in Table 1.

**Table 1:** Physicochemical Properties of ZIF-8 and APA@ZIF-8 Nanoparticles, Including Yield, Hydrodynamic Size, Drug Loading cCapacity, Encapsulation Efficiency, and Zeta Potential

	Yield (%)	Hydrodynamic radius (nm)	Zeta Potential (mV)	Drug Loading Capacity (%)	Drug Encapsulation Efficiency (%)
ZIF-8	68.3	340	21.3		
APA@ZIF-8	69.4	200	24.5	47.8	69.4

Morphological characterization by SEM showed well-defined rhombic dodecahedral particles with sizes ranging from approximately 114 to 200 nm (Figure 1B), consistent with typical ZIF-8 crystal structures reported in the literature.^38^ STEM analysis further confirmed that particle morphology and structural integrity were largely preserved after drug loading, indicating that the encapsulation process did not significantly affect the framework. The retention of morphology suggests that the robust coordination structure of ZIF-8 remains intact during drug incorporation, supporting its suitability as a drug delivery system. Additionally, this observation implies that drug molecules are likely accommodated within the internal pore structure rather than being limited to surface adsorption. Although minor aggregation was observed, this is common in nanoscale systems due to high surface energy and interparticle interactions. Such aggregation may affect dispersion stability and should be considered in future studies, where surface modification strategies could be employed to improve colloidal stability.

Dynamic light scattering (DLS) analysis revealed that the hydrodynamic diameter decreased from 340 nm for bare ZIF-8 to 200 nm after APA loading, indicating structural compaction upon drug incorporation (Figure 1C). The zeta potential of the nanoparticles changed from −21.3 mV for ZIF-8 to +24.5 mV for APA@ZIF-8 (Table 1; Figure 1D), indicating a pronounced alteration in surface charge after APA loading. This shift suggests an interaction between apalutamide and the ZIF-8 framework and reflects changes in the surface characteristics of the nanoparticles. Since ZIF-8 is a well-established metal–organic framework with widely reported physicochemical properties,^39,40^ the observed difference in zeta potential is likely associated with drug incorporation as well as the specific composition of the synthesized nanosystem and the measurement conditions. Overall, these results demonstrate that APA was successfully encapsulated within ZIF-8 while maintaining structural integrity, favorable particle size, and surface properties suitable for biomedical applications.

Energy-dispersive X-ray (EDX) elemental mapping further confirmed the homogeneous distribution of the framework-related elements, including Zn, C, and N, throughout the individual crystals, supporting the formation of a structurally uniform ZIF-8 matrix. In addition, the presence of F and S signals in the APA@ZIF-8 sample provided clear evidence for the successful incorporation of apalutamide, as these elements are characteristic of the drug structure (Figure 1E).

The uniform spatial distribution of F and S signals suggests that the drug is well-dispersed within the framework rather than being localized only on the external surface, which is advantageous for achieving consistent drug loading and controlled release behavior. Furthermore, the absence of noticeable elemental segregation indicates that the encapsulation process did not disrupt the compositional integrity of the system. Overall, the elemental mapping results not only verify successful drug incorporation but also highlight the compositional homogeneity of the nanosystem, which is a critical factor for reproducibility and performance in drug delivery applications.

The crystallinity and phase purity of the synthesized ZIF-8 and APA@ZIF-8 nanoparticles were confirmed by powder X-ray diffraction analysis. The experimental diffraction patterns were consistent with the simulated pattern derived from reported ZIF-8 structural data, indicating the successful formation of pure-phase ZIF-8 (Figure 1F). The PXRD patterns exhibited characteristic diffraction peaks within the 2θ range of 5–40°, corresponding to the well-defined porous crystalline structure of ZIF-8. Prominent reflections observed at 2θ = 7.11°, 12.5°, 17.75°, and 26.4° were indexed to the (011), (002), (112), (022), (013), and (222) planes, in agreement with previously reported data.^41,42^ The slight broadening of the diffraction peaks suggests the formation of nanoscale crystalline domains. The calculated interplanar spacings, based on Bragg’s law, were consistent with the typical sodalite-type framework of ZIF-8, exhibiting a body-centered cubic structure with a unit cell parameter of approximately 17 Å.^43^ These results confirm the high crystallinity and structural integrity of the synthesized nanoparticles. Importantly, the PXRD pattern of APA@ZIF-8 retained all characteristic peaks of pristine ZIF-8 without any noticeable shifts or additional peaks (Figure S1), indicating that the incorporation of apalutamide did not alter the crystal structure of the framework. This suggests that APA was successfully encapsulated within the pores of ZIF-8 without disrupting its lattice structure or inducing any secondary phase formation.

FTIR spectra of ZIF-8 and APA@ZIF-8 are presented in Figure 1G. The spectrum of ZIF-8 exhibited the characteristic vibrational bands of the framework, including aromatic and aliphatic C–H stretching at 3135 and 2928 cm^–1^, bands at 1606 and 1580 cm^–1^ attributed to C=C/C–N stretching, the C–N absorption region at 1100–1400 cm^–1^, and the Zn–N vibration at 421 cm^–1^, consistent with previous reports.^41^ Compared with pristine ZIF-8, the FTIR spectrum of APA@ZIF-8 did not show distinct additional bands, which is likely due to the overlap of APA-related vibrations with the characteristic absorption bands of the ZIF-8 framework. This masking effect is commonly observed in drug-loaded MOF systems and suggests that the encapsulated drug is confined within the porous structure. No new peaks were detected in the APA@ZIF-8 spectrum, indicating that no covalent bond formation occurred between APA and the ZIF-8 framework. Instead, the interaction is likely governed by noncovalent forces such as hydrogen bonding and π–π interactions. Minor variations in peak intensity and sharpness further support the presence of weak interactions between APA and the framework. Overall, the FTIR results indicate that APA was successfully incorporated into the ZIF-8 structure without altering its chemical framework. This interpretation is consistent with the PXRD results, which confirmed the preservation of crystallinity after drug loading. Therefore, FTIR analysis, together with complementary characterization techniques, supports the successful encapsulation of APA within the ZIF-8 nanosystem.

Thermogravimetric analysis (TGA) was performed to evaluate the thermal stability and drug incorporation of ZIF-8 and APA@ZIF-8 nanoparticles under a nitrogen atmosphere (Figure 2A). Both samples exhibited minimal weight loss below 130 °C, which can be attributed to the removal of physically adsorbed moisture. A significant difference between ZIF-8 and APA@ZIF-8 was observed at elevated temperatures. While pristine ZIF-8 showed negligible weight loss (∼0.64%) at around 200 °C, APA@ZIF-8 exhibited a higher weight loss (∼9%), indicating the presence of encapsulated apalutamide within the framework. This additional mass loss is attributed to the thermal decomposition of the drug molecules confined within the porous structure. At higher temperatures (200–400 °C), the difference in weight loss between the two samples persisted, further supporting successful drug incorporation. Above this range, both samples exhibited similar decomposition profiles, corresponding to the degradation of the ZIF-8 framework. A major weight loss was observed beyond ∼600 °C, associated with the collapse of the crystalline structure and the formation of residual inorganic species.^43^ Overall, the distinct thermal behavior of APA@ZIF-8 compared to pristine ZIF-8 confirms the successful encapsulation of apalutamide and demonstrates that the nanocarrier maintains good thermal stability.

**Figure 2:**
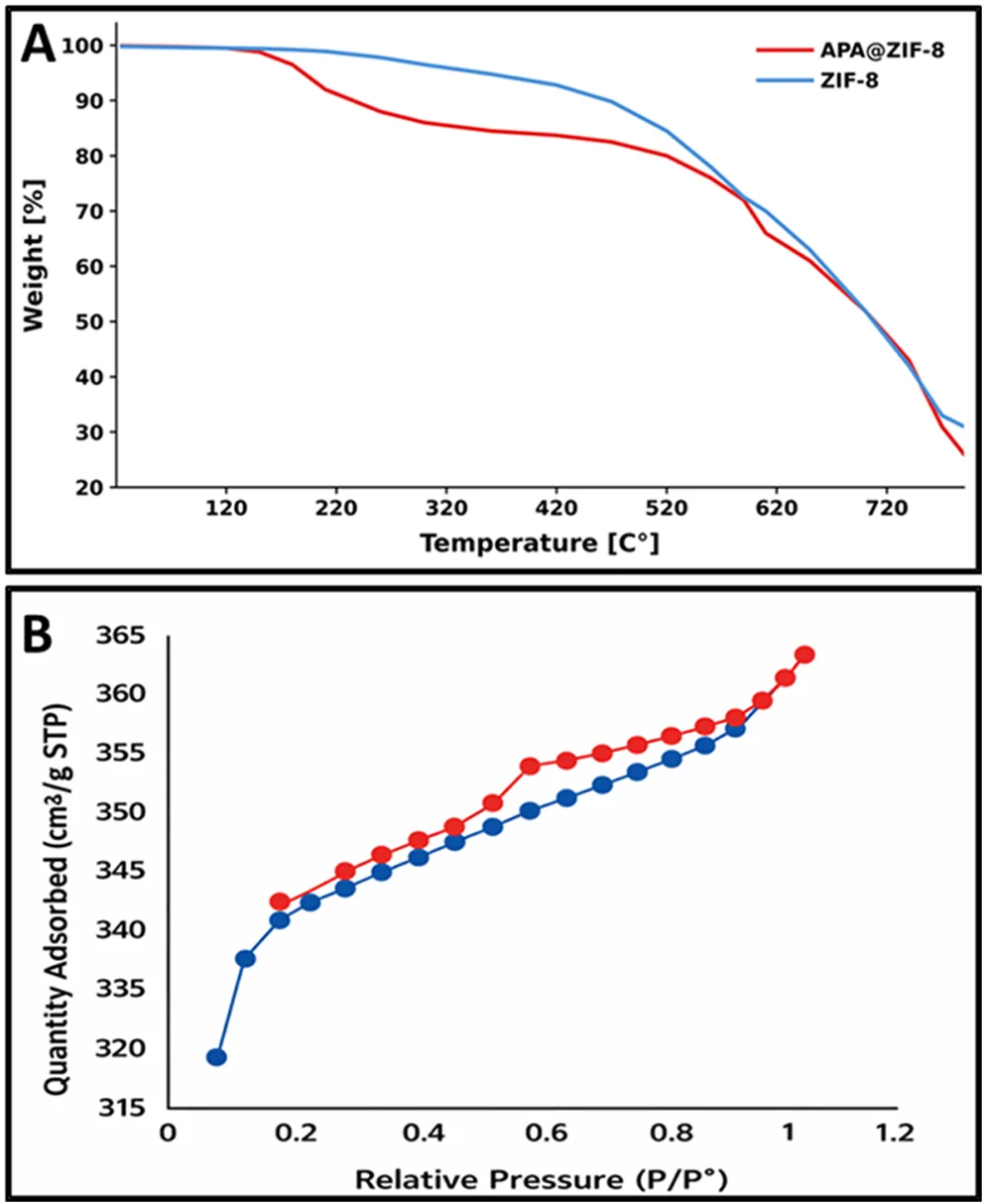
(A) TGA curves of ZIF-8 and APA@ZIF-8. (B) N_2_ adsorption–desorption isotherms of the ZIF-8.

The nitrogen adsorption–desorption isotherms of ZIF-8 nanoparticles are shown in Figure 2B. The isotherm exhibited a typical Type I profile, characteristic of microporous materials. The Brunauer–Emmett–Teller (BET) surface area and Langmuir surface area were determined to be 1017.32 m^2^ g^–1^ and 1495.72 m^2^ g^–1^, respectively. These values are consistent with previously reported data for ZIF-8,^44^ confirming the preservation of its porous structure after synthesis. The high surface area and microporous nature of ZIF-8 are critical for efficient drug loading and controlled release, supporting its suitability as a nanocarrier system.

The in vitro drug release behavior of APA@ZIF-8 nanoparticles was evaluated under different pH conditions to simulate physiological (pH 7.4) and acidic tumor/endosomal environments (pH 5.0). The release profiles were obtained by incubating the nanoparticles in PBS buffer at 37 °C under gentle agitation, and the released apalutamide was quantified using UV–vis spectroscopy (Figure 3). APA@ZIF-8 exhibited a clear pH-dependent release behavior, with significantly faster drug release observed under acidic conditions compared to physiological pH. At pH 5.0, a rapid initial release was followed by a sustained release phase, reaching a cumulative release of approximately 93% over 120 h. In contrast, at pH 7.4, a slower and more controlled release profile was observed, with approximately 62% drug release over the same period. The enhanced drug release at acidic pH can be attributed to the pH-sensitive degradation of the ZIF-8 framework. Under neutral conditions (pH 7.4), ZIF-8 remains relatively stable due to strong coordination between Zn^2+^ ions and imidazolate linkers. However, under acidic conditions, protonation of the imidazolate ligands weakens Zn–N coordination bonds, leading to gradual framework disintegration and accelerated drug release. An initial burst release was observed during the early stage, which can be attributed to the release of drug molecules adsorbed near or on the surface of the nanoparticles. This was followed by a sustained release phase, indicating that the majority of apalutamide was encapsulated within the porous structure of ZIF-8. The relatively slower release at physiological pH is advantageous for minimizing premature drug leakage during systemic circulation, while the accelerated release under acidic conditions supports targeted drug delivery within tumor or intracellular environments. These findings confirm that APA@ZIF-8 functions as a pH-responsive nanocarrier, enabling controlled and site-specific drug release.

**Figure 3:**
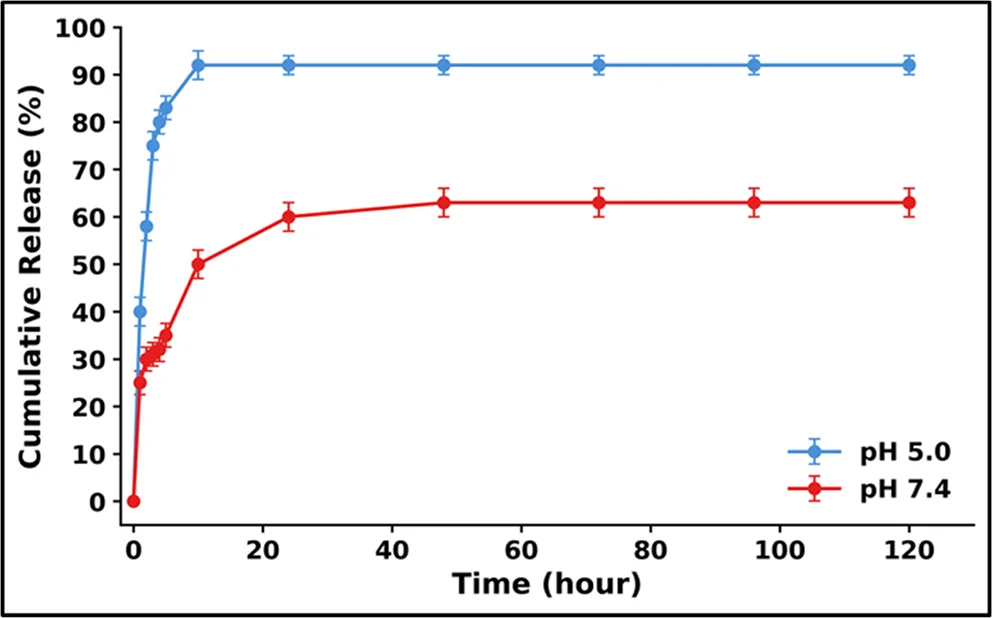
In vitro drug release profiles of APA@ZIF-8 nanoparticles at pH 5.0 and 7.4. Data are presented as mean ± SD (*n* = 3).

To further support the release mechanism, PXRD analysis was performed after incubation under different pH conditions (Figure S1). A gradual decrease in the intensity of characteristic ZIF-8 diffraction peaks, particularly the (011) plane, was observed with increasing incubation time, indicating structural degradation of the framework. This effect was more pronounced under acidic conditions, confirming that the accelerated drug release is directly associated with pH-induced destabilization of the ZIF-8 structure. Overall, the results demonstrate that APA@ZIF-8 provides a controlled and pH-responsive drug release profile, which is highly desirable for improving therapeutic efficacy while reducing off-target effects in prostate cancer treatment.

Serum albumin is the most abundant plasma protein and plays a critical role in drug transport, distribution, and pharmacokinetics. The interaction between drug-loaded nanoparticles and serum proteins can significantly influence circulation time, bioavailability, and therapeutic efficacy. Drugs may exist in the bloodstream in either bound or unbound forms, where only the unbound fraction is considered pharmacologically active. In this study, serum protein binding of APA, ZIF-8, and APA@ZIF-8 nanoparticles were evaluated using FBS, and the binding percentages were calculated based on the unbound protein remaining in the supernatant. As presented in Table 2, protein binding did not show a linear correlation with increasing serum ratios, suggesting complex interaction dynamics between nanoparticles and serum proteins. Compared to literature reports where nanoparticle–protein binding can reach up to ∼ 40%, the binding percentages observed in this study remained relatively moderate. Notably, APA@ZIF-8 exhibited the highest binding at 60:40 ratio (24.85 ± 2.74%), while free APA showed significantly lower interaction. This indicates that encapsulation within the ZIF-8 framework enhances protein interaction without leading to excessive binding. The moderate protein binding observed for APA@ZIF-8 suggests a favorable balance between circulation stability and bioavailability. Excessive protein binding may reduce therapeutic efficacy, whereas very low binding may lead to rapid clearance. Therefore, the obtained results indicate that APA@ZIF-8 nanoparticles possess suitable protein interaction characteristics for effective systemic delivery and potential accumulation in target tissues, supporting their biocompatibility and therapeutic applicability. At the other serum-to-nanocarrier ratios, protein binding percentages were lower for both the drug-loaded and drug-free nanoparticles.^45,46^

**Table 2:** Protein Binding Percentages of APA, ZIF-8, and APA@ZIF-8 (n.d.: Not Determined)

Sample	*V*_FBS_:*V*_Sample_	Protein Binding %
APA	10:90	n.d.
	20:80	n.d.
	30:70	n.d.
	40:60	n.d.
	50:50	4.12 ± 0.83
	60:40	7.68 ± 1.95
	70:30	10.94 ± 2.12
	80:20	9.37 ± 1.76
	90:10	8.21 ± 1.44
ZIF-8	10:90	n.d.
	20:80	n.d.
	30:70	n.d.
	40:60	n.d.
	50:50	11.28 ± 3.45
	60:40	18.73 ± 4.12
	70:30	16.42 ± 3.98
	80:20	14.87 ± 3.56
	90:10	13.95 ± 2.88
AP@ZIF-8	10:90	n.d.
	20:80	n.d.
	30:70	n.d.
	40:60	n.d.
	50:50	8.96 ± 2.21
	60:40	25.32 ± 1.65
	70:30	13.72 ± 2.65
	80:20	10.58 ± 2.31
	90:10	7.94 ± 1.82

Hemocompatibility of ZIF-8 and APA@ZIF-8 nanoparticles were evaluated using an in vitro hemolysis assay. Since erythrocytes are among the first biological components encountered by intravenously administered nanoparticles, assessing hemolytic activity is essential for determining blood compatibility. As shown in Figure 4, both ZIF-8 and APA@ZIF-8 nanoparticles exhibited very low hemolysis percentages at all tested concentrations (2, 10, and 20 μg/mL). The hemolysis values were determined to be 0.53% for ZIF-8 and 0.54% for APA@ZIF-8, indicating negligible membrane disruption. According to the ASTM F-756–08 standard, materials are classified as nonhemolytic when the hemolysis percentage is below 2%. In this study, all measured values were well below the 5% threshold and remained within the nonhemolytic range.^47^ These results demonstrate that both ZIF-8 and APA@ZIF-8 nanoparticles exhibit excellent hemocompatibility. Importantly, the encapsulation of apalutamide within the ZIF-8 framework did not induce additional hemolytic effects, confirming that the developed nanocarrier system is safe for blood-contact applications and suitable for systemic administration.

**Figure 4:**
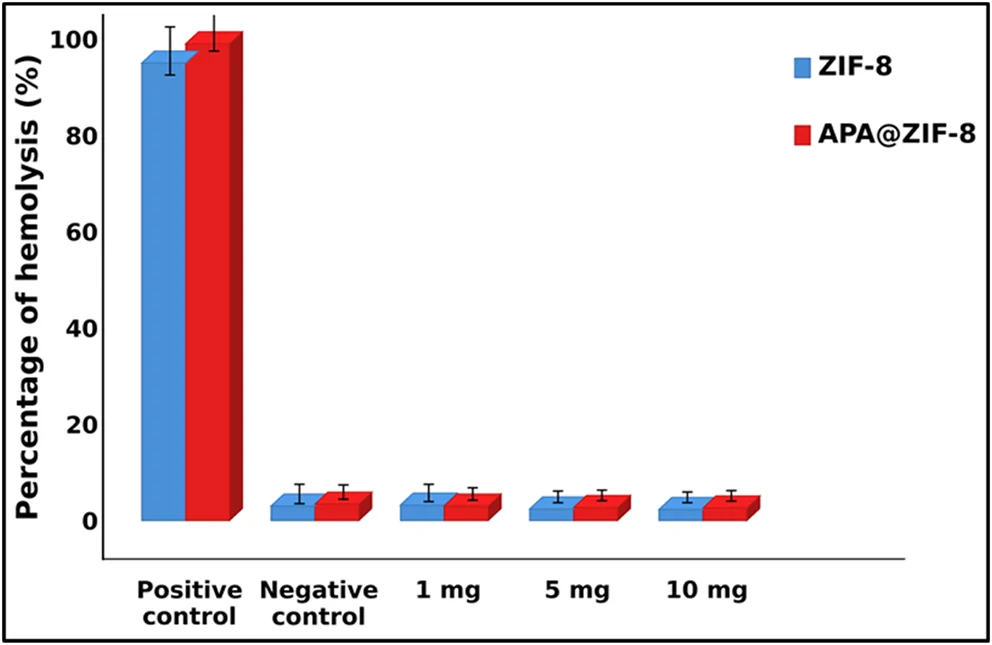
Hemolysis rates of ZIF-8 and APA@ZIF-8 particles at 1 mg, 5 mg and 10 mg. Data are presented as mean ± SD (*n* = 3).

The cytotoxic effects of ZIF-8 and APA@ZIF-8 nanoparticles were evaluated using the WST-1 assay in LNCaP, 104R2, and PC-3 prostate cancer cell lines at different concentrations (25–250 μg mL^–1^) and incubation times (24, 48, and 72 h) (Figure 5). The cytotoxic activity of APA was also evaluated in LNCaP, PC3, and 104R2 cell lines at 24, 48, and 72 h (Figure S2). The results indicate that ZIF-8 exhibited relatively low cytotoxicity across all tested cell lines, confirming its biocompatible nature. At lower concentrations (25 μg mL^–1^), a slight increase in cell viability was observed, which may be attributed to the release of Zn^2+^ ions that can participate in cellular metabolic processes. However, at higher concentrations, a moderate decrease in viability was detected, likely due to Zn^2+^-induced oxidative stress and mitochondrial effects, as reported in the literature.^48^ In contrast, APA@ZIF-8 nanoparticles demonstrated a clear concentration- and time-dependent cytotoxic effect in all prostate cancer cell lines. In LNCaP cells (Figure 5A), no significant reduction in viability was observed at 24 h, which is consistent with the controlled release profile of apalutamide. However, at 48 and 72 h, a marked decrease in cell viability (approximately 15–30%) was observed, particularly at higher concentrations (100 and 250 μg mL^–1^), indicating enhanced cytotoxic activity over time. Similarly, APA@ZIF-8 exhibited dose- and time-dependent cytotoxicity in 104R2 cells (Figure 5B), although the response was less pronounced compared to LNCaP and PC-3 cells. This reduced sensitivity may be associated with the androgen-independent nature of the 104R2 cell line. In PC-3 cells (Figure 5C), both ZIF-8 and APA@ZIF-8 affected cell viability in a concentration-dependent manner; however, APA@ZIF-8 showed significantly stronger antiproliferative effects. Notably, concentrations above 25 μg mL^–1^ led to a substantial reduction in cell viability, with the most pronounced effect observed at 250 μg mL^–1^. The progressive decrease in viability over time suggests sustained drug release and prolonged intracellular activity. The enhanced cytotoxicity of APA@ZIF-8 compared to ZIF-8 can be attributed to the combined effects of apalutamide and Zn^2+^ ions released from the framework. The delayed but sustained reduction in cell viability is consistent with the pH-responsive release behavior and PXRD findings, which indicated gradual structural degradation of ZIF-8 over time. Overall, the results demonstrate that APA@ZIF-8 induces a time- and dose-dependent antiproliferative effect in prostate cancer cells, while ZIF-8 alone exhibits minimal cytotoxicity. These findings support the potential of APA@ZIF-8 as a dual-function nanotherapeutic system combining drug delivery with zinc-mediated cytotoxicity. Further studies on normal cell lines are required to evaluate its therapeutic selectivity and biocompatibility.

**Figure 5:**
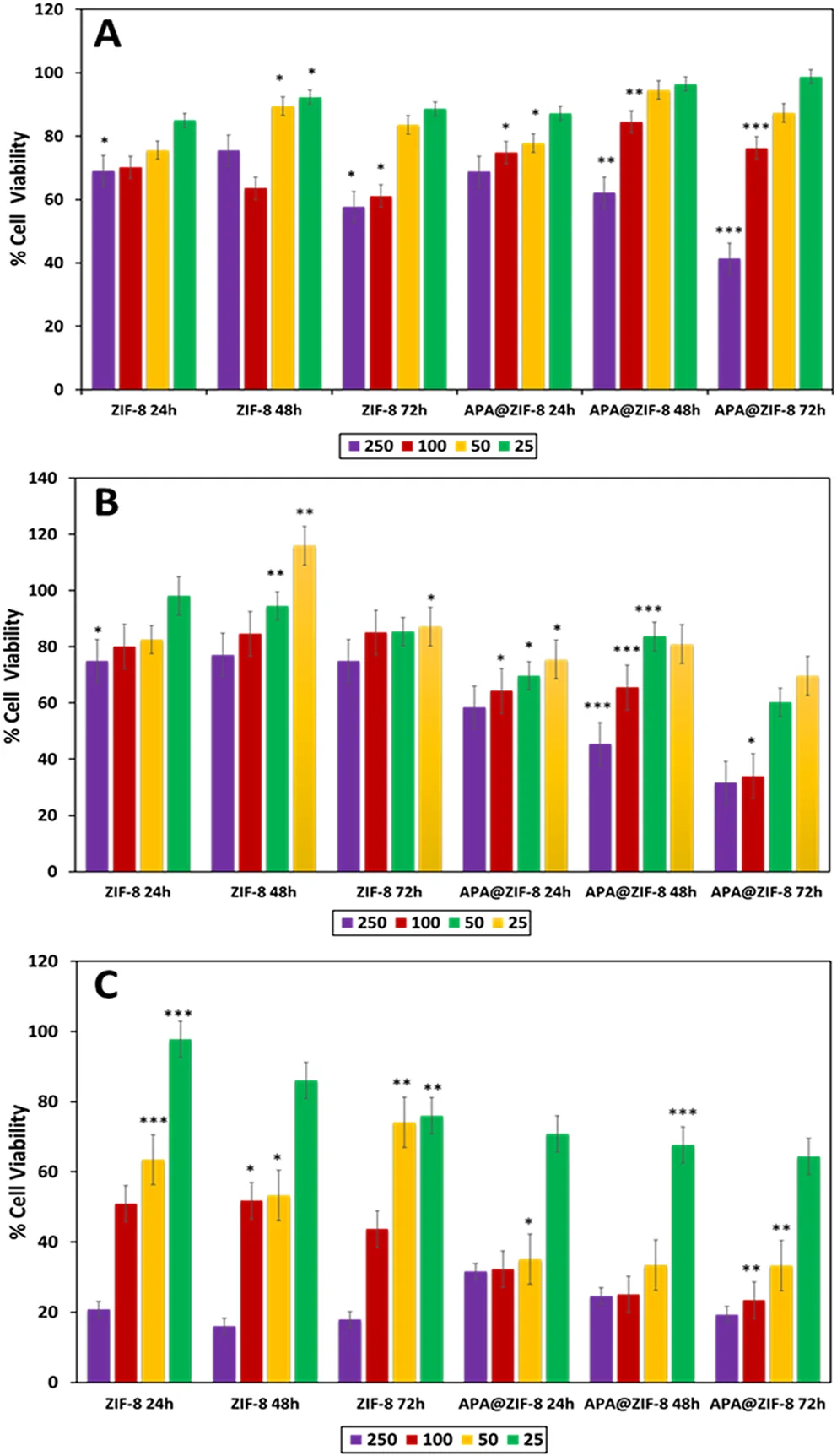
*In vitro* cytotoxicity profile of ZIF-8 and APA@ZIF-8 against (A**)** LnCaP, (B**)** 104R2, and (C) PC3 cell lines at 24, 48, and 72 h incubation time as assayed by MTT at various concentrations (25–250 μg/mL). Data are presented as mean ± SD (*n* = 3). Statistical analysis was performed using two-way ANOVA followed by Tukey’s multiple comparisons test. *p* < 0.05, *p* *<* 0.01, and *p* *<* 0.001 versus the untreated control group.

The apoptotic effects of ZIF-8 and APA@ZIF-8 nanoparticles on LNCaP, PC3, and 104R2 prostate cancer cell lines were evaluated using Annexin V-FITC/PI staining. As shown in Figure 6A, treatment with APA@ZIF-8 resulted in a clear increase in both early and late apoptotic cell populations compared to the control and ZIF-8-treated groups in all examined cell lines. This trend was particularly evident in LNCaP and PC3 cells, indicating that the drug-loaded nanosystem exerts a stronger pro-apoptotic effect than the blank carrier. In contrast, ZIF-8 alone induced only limited apoptotic changes, supporting its relatively low intrinsic cytotoxicity under the tested conditions. The enhanced apoptotic response observed after APA@ZIF-8 treatment is likely associated with the combined action of intracellular apalutamide release and Zn^2+^-mediated oxidative stress, which together promote apoptosis-related signaling pathways. To further investigate whether oxidative stress was associated with perturbation of cell cycle progression, flow cytometric cell cycle analysis was performed using propidium iodide (PI) staining (Figure 6B). The distribution of cells across the G0/G1, S, and G2/M phases was analyzed in PC3 and 104R2 cells after treatment with 10 μg mL^–1^ ZIF-8. As shown in Figure 6B, no significant alterations were observed in cell cycle phase distribution relative to the untreated control. These findings suggest that ZIF-8 alone, at the tested concentration, does not markedly interfere with cell cycle progression. Taken together, the apoptosis and cell cycle results indicate that the blank carrier is largely biocompatible, whereas the APA-loaded nanosystem induces a stronger cytotoxic response primarily through apoptosis rather than through major nonspecific disruption of cell cycle regulation.

**Figure 6:**
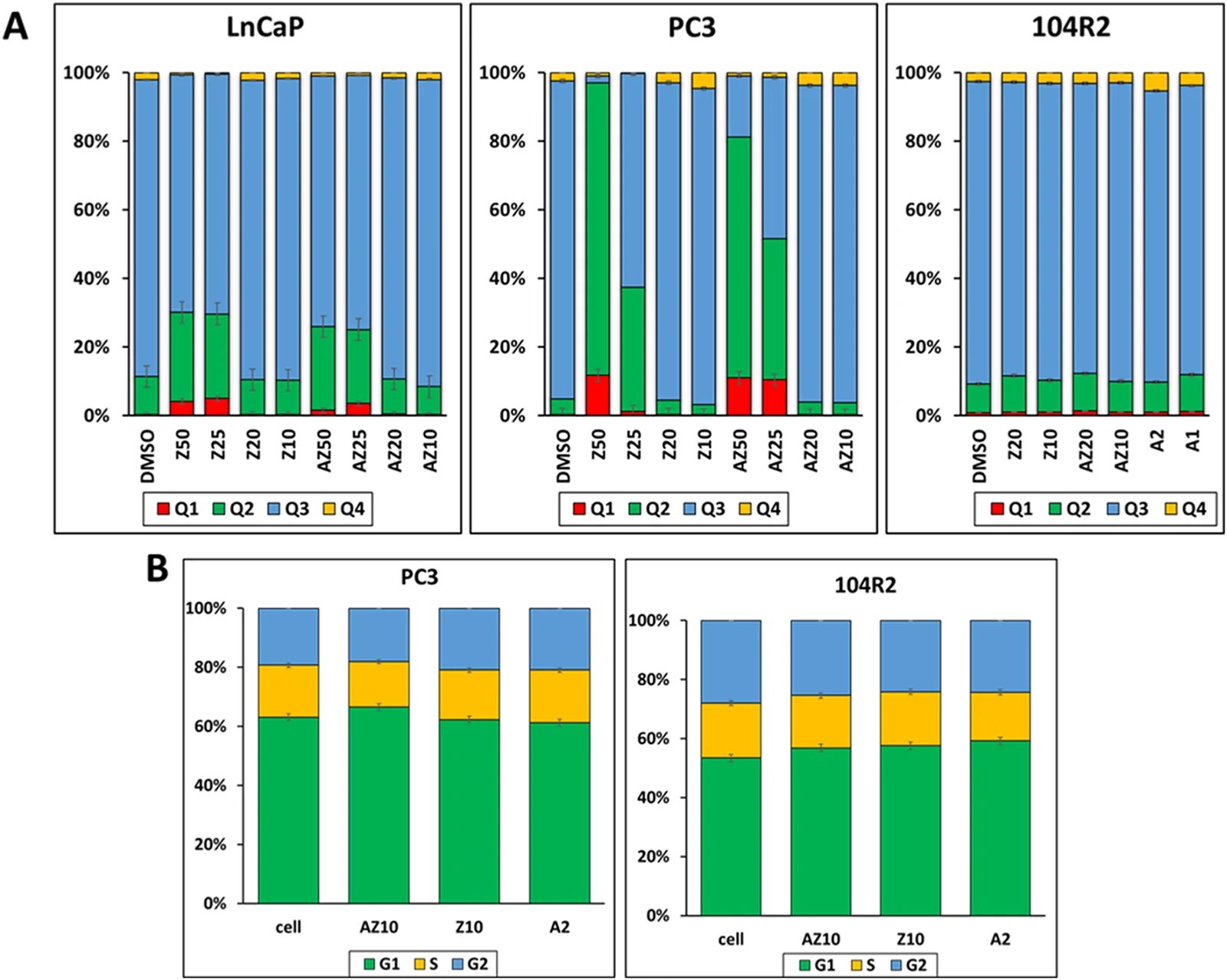
(A) Apoptosis analysis of ZIF-8 and APA@ZIF-8 in LNCaP, PC3, and 104R2 cell lines after 24 h of treatment. Quadrants represent necrosis (Q1), late apoptosis (Q2), viable cells (Q3), and early apoptosis (Q4). (B) Cell cycle analysis of PC3 and 104R2 cell lines after treatment with 10 μg mL^–1^ ZIF-8 for 24 h. DNA content was quantified by propidium iodide (PI) staining, and the distribution of cells in the G0/G1, S, and G2/M phases was analyzed by flow cytometry. Data are presented as mean ± SD (*n* = 3).

Intracellular reactive oxygen species (ROS) generation was assessed using the DCFH-DA assay (Figure 7A). All treated groups exhibited higher fluorescence intensity than the control, indicating increased ROS production. Among them, APA@ZIF-8-treated cells displayed the highest ROS levels, whereas free apalutamide induced comparatively lower ROS generation. This finding demonstrates that the nanosystem markedly enhances intracellular oxidative stress. Such an effect is likely related to the efficient cellular uptake of ZIF-8-based nanoparticles followed by pH-responsive intracellular degradation, which enables the release of both apalutamide and Zn^2+^ ions. Since elevated intracellular zinc levels are known to impair mitochondrial function and amplify oxidative stress, the observed ROS enhancement is consistent with the proposed dual-action mechanism of the nanosystem

**Figure 7:**
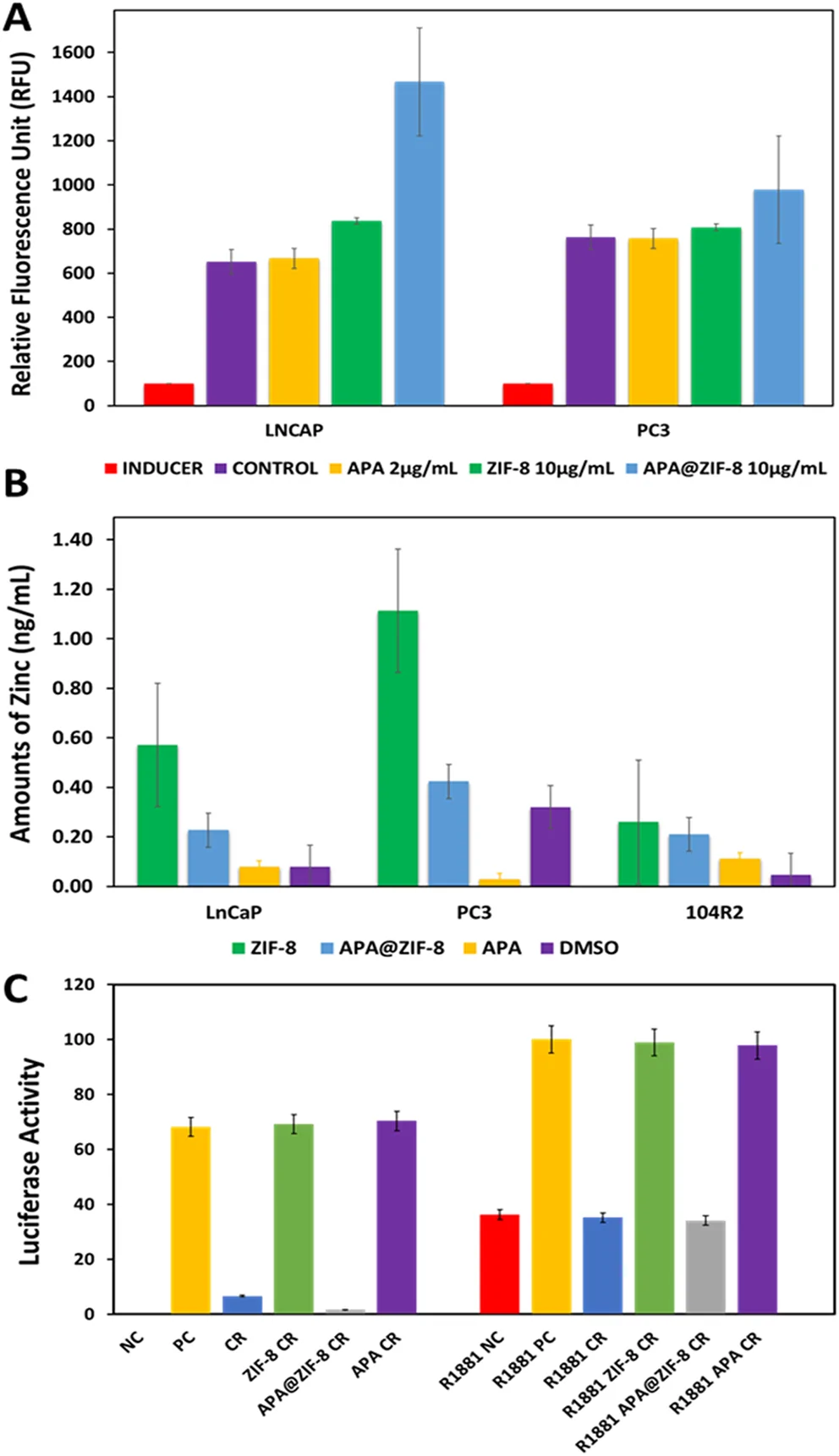
(A) Intracellular reactive oxygen species (ROS) levels in LNCaP and PC3 cell lines after 24 h of treatment with ZIF-8, APA, and APA@ZIF-8, measured using the DCFH-DA assay. Fluorescence intensity reflects intracellular ROS generation. (B) Quantification of intracellular Zn^2+^ levels in LNCaP, PC3, and 104R2 cell lines after 24 h of treatment with ZIF-8, APA@ZIF-8, APA, and DMSO (control). (C) Luciferase reporter assay showing androgen receptor (AR) transcriptional activity in the presence and absence of R1881 after treatment with ZIF-8, APA, and APA@ZIF-8. Data are presented as mean ± SD (*n* = 3). Statistical analysis was performed using one-way ANOVA followed by Tukey’s post hoc test (*p < 0.05, **p < 0.01, ***p < 0.001).

Intracellular zinc levels in LNCaP, PC3, and 104R2 prostate cancer cells after 24 h of treatment with ZIF-8, APA@ZIF-8, APA, and DMSO (control) are shown in Figure 7B. Due to their nanoscale size, ZIF-8-based particles are expected to be internalized through endocytic pathways.^49^ Because prostate cancer cells typically exhibit reduced intracellular zinc levels,^50^ treatment-induced zinc accumulation could be readily detected. As shown in Figure 7B, intracellular zinc levels differed among the tested cell lines, with the highest levels generally observed after treatment with 10 μg mL^–1^ ZIF-8 or APA@ZIF-8. These findings indicate that both formulations enhance intracellular zinc accumulation. Increased intracellular Zn^2+^ levels have been associated with mitochondrial dysfunction and oxidative stress^51^ which may help explain the elevated ROS levels observed in this study. In LNCaP cells, ZIF-8 treatment induced an approximately 6-fold increase in intracellular zinc, whereas APA@ZIF-8 induced an approximately 3-fold increase. In PC3 cells, the increase following APA@ZIF-8 treatment was comparatively lower. These differences likely reflect cell line-dependent variations in nanoparticle uptake, intracellular trafficking, or zinc homeostasis. Overall, these results support the role of ZIF-8-based nanoparticles as effective intracellular zinc delivery systems.

The effect of synthetic androgen R1881 on androgen receptor (AR) localization was investigated in LNCaP cells. As shown in Figure 7C, treatment with R1881 induced translocation of AR from the cytoplasm to the nucleus, a characteristic feature of AR activation. In the absence of nanoparticle treatment, AR was predominantly localized in the nucleus after 24 h of R1881 exposure, confirming the androgen-responsive behavior of LNCaP cells. In the presence of ZIF-8, AR localization was partially altered, with a fraction of the receptor remaining in the cytoplasm, suggesting a limited modulatory effect. In contrast, treatment with APA@ZIF-8 resulted in a more pronounced disruption of AR nuclear localization, indicating more effective interference with AR signaling. Compared with conventional antiandrogens such as bicalutamide, apalutamide has been reported to exhibit a distinct mode of AR inhibition, which may partly account for the observed differences in AR translocation behavior.^52^ Collectively, these findings are consistent with the well-established mechanism of AR activation, in which ligand binding induces conformational changes, nuclear import, and transcriptional activation. Although additional high-resolution imaging approaches may further clarify the subcellular dynamics of AR localization, the present results provide clear evidence that APA@ZIF-8 interferes with androgen-induced AR nuclear translocation in LNCaP cells.

To further evaluate the functional consequences of AR modulation, a luciferase reporter assay was performed to assess AR transcriptional activity. In the absence of R1881, neither ZIF-8 nor APA significantly affected AR transcriptional activity, as the measured levels were comparable to those of the control group. Upon R1881 stimulation, luciferase activity increased markedly in all groups, confirming activation of AR signaling. Notably, APA@ZIF-8 treatment produced a significant reduction in luciferase activity even under androgen-stimulated conditions, whereas ZIF-8 and free APA showed comparatively limited inhibitory effects. This enhanced suppression of AR transcriptional activity is likely attributable to improved intracellular delivery and sustained release of apalutamide from the ZIF-8 framework. Taken together, these findings demonstrate that APA@ZIF-8 effectively interferes with both AR nuclear localization and AR-dependent transcriptional activity, highlighting its potential as a nanotherapeutic platform for enhanced inhibition of androgen receptor signaling in prostate cancer cells.

Taken together, the combined findings from ROS analysis, intracellular zinc quantification, and AR reporter assays indicate that APA@ZIF-8 exerts its anticancer effects through multiple complementary pathways. In addition to improving drug delivery, the nanosystem promotes intracellular zinc accumulation and oxidative stress while simultaneously suppressing AR transcriptional activity. This multimodal mechanism is particularly relevant in prostate cancer, where both ROS-mediated cytotoxicity and inhibition of AR signaling may contribute to improved therapeutic efficacy.

## 4 Conclusions

In this study, a pH-responsive metal–organic framework-based nanocarrier system (APA@ZIF-8) was successfully developed for the enhanced treatment of prostate cancer. The nanosystem was designed to integrate the therapeutic effects of apalutamide, a clinically approved androgen receptor (AR) inhibitor, with the intrinsic biological activity of zinc ions released from the ZIF-8 framework. The one-pot synthesis approach enabled efficient encapsulation of apalutamide, resulting in high drug loading capacity and encapsulation efficiency, while preserving the structural integrity and characteristic morphology of the ZIF-8 framework. Comprehensive physicochemical characterization confirmed the successful formation of the nanosystem. The obtained nanoparticles exhibited well-defined morphology, nanoscale size distribution, and preserved crystalline structure after drug loading. Importantly, the incorporation of apalutamide did not disrupt the framework architecture, indicating that the drug was effectively confined within the porous structure of ZIF-8. The in vitro drug release studies demonstrated a clear pH-dependent release profile, with significantly accelerated drug release under acidic conditions compared to physiological pH. This behavior is attributed to the pH-sensitive degradation of the ZIF-8 framework, which facilitates selective drug release in tumor microenvironments and intracellular compartments. Such a release profile is highly advantageous for minimizing premature drug leakage during circulation while enabling targeted therapeutic action at the disease site. Biological evaluations revealed that APA@ZIF-8 exhibits enhanced anticancer activity compared to both ZIF-8 and free apalutamide. The nanosystem induced a concentration- and time-dependent reduction in cell viability across multiple prostate cancer cell lines, including LNCaP, PC3, and 104R2. While ZIF-8 alone showed minimal cytotoxicity, its ability to deliver zinc intracellularly contributed to the overall therapeutic effect of the combined system. Mechanistic studies further demonstrated that APA@ZIF-8 significantly enhances intracellular reactive oxygen species (ROS) generation, which is associated with zinc-mediated mitochondrial dysfunction and oxidative stress. In addition, the nanosystem was shown to interfere with androgen receptor signaling by modulating AR localization and effectively suppressing AR transcriptional activity, even under androgen-stimulated conditions. These findings indicate that APA@ZIF-8 operates through a dual therapeutic mechanism involving both hormonal pathway inhibition and metal ion-induced cytotoxicity. Overall, the integration of controlled drug delivery with zinc-mediated intracellular effects provides a synergistic therapeutic strategy that may overcome limitations associated with conventional prostate cancer treatments. The developed APA@ZIF-8 nanosystem represents a multifunctional and biocompatible platform with significant potential for targeted cancer therapy. Although the present study demonstrates promising in vitro results, further investigations are required to fully evaluate the clinical applicability of this system. Future studies should focus on in vivo validation, including pharmacokinetics, biodistribution, tumor targeting efficiency, and long-term safety. In addition, optimization of dosage, surface functionalization, and evaluation in more complex biological models will be essential for advancing this nanoplatform toward translational and clinical applications.

## Supplementary Material



## Data Availability

All data supporting the findings of this study are available within the manuscript.

## Notes

Additional PXRD analyses showing the structural evolution of ZIF-8 and APA@ZIF-8 nanoparticles after incubation in PBS at pH 3 and pH 5, and cytotoxicity profiles of free apalutamide (APA) in LNCaP, PC3, and 104R2 cell lines at 24, 48, and 72 h (PDF)
