# Supplementary material for: pH-Responsive Apalutamide-Loaded ZIF‑8 Nanoparticles for Zinc-Enhanced Dual Cytotoxicity in Prostate Cancer
Source: ACS Omega. 2026 Aug 17;11(34):50818–30. doi: 10.1021/acsomega.6c01591 (PMC13625125; doi:10.1021/acsomega.6c01591)
Supplement: Supplementary file 1 [file ao6c01591_si_001.pdf]

pH-Responsive Apalutamide-Loaded ZIF-8 Nanoparticles for Zinc-Enhanced Dual Cytotoxicity in Prostate Cancer

Derya Mete and Gülşah Şanlı-Mohamed*

İzmir Institute of Technology, Department of Chemistry, İzmir, 35430, Türkiye

***Corresponding Author**

Prof. Dr. Gülşah Şanlı-Mohamed
Izmir Institute of Technology, Department of Chemistry, İzmir, 35430, Türkiye
Phone: +90 2327507515; Fax: +90 2327507509
E-mail: gulsahsanli@iyte.edu.tr, gulsahsanli@gmail.com

Supplementary Data

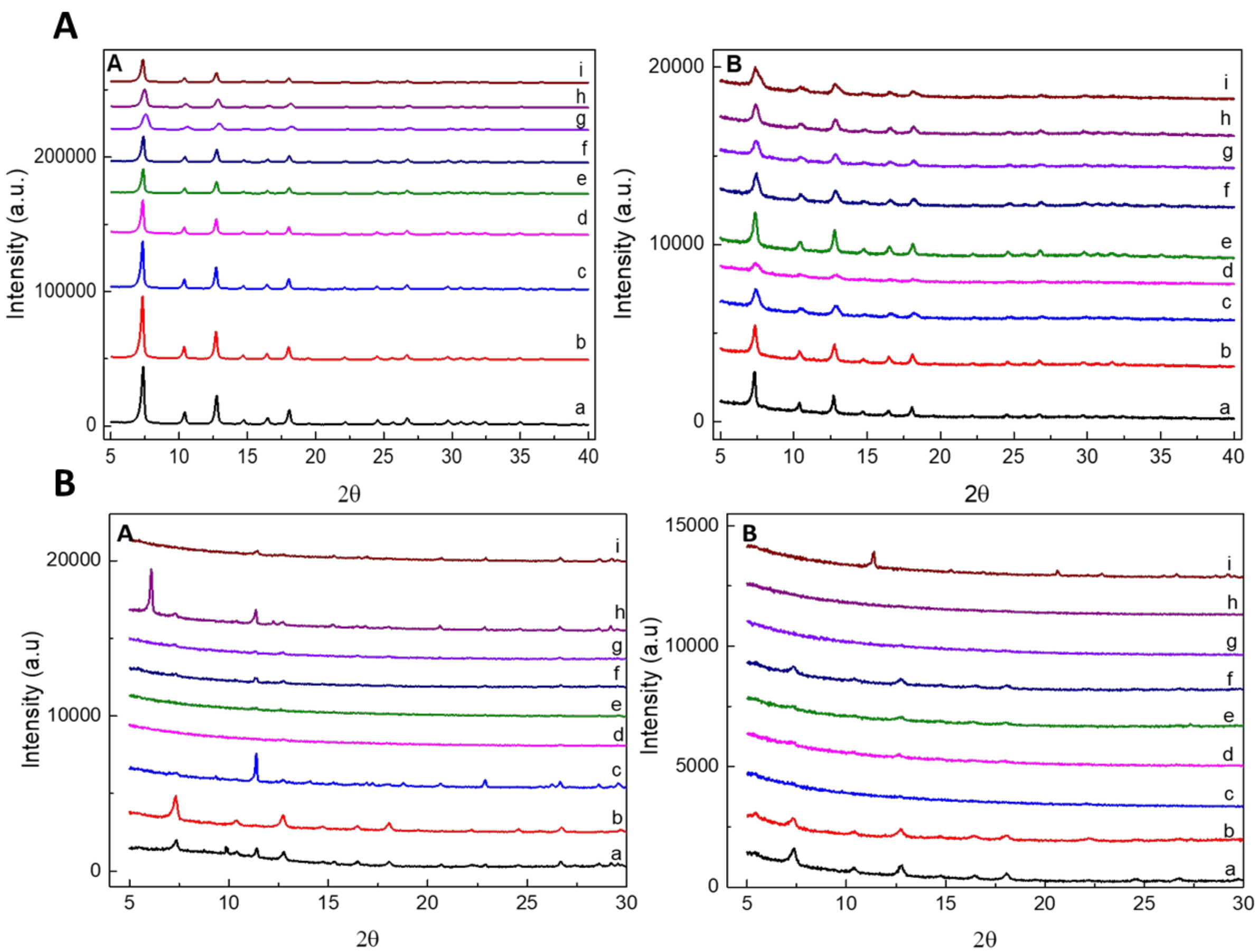

Figure S1. (A) PXRD patterns illustrating the structural evolution of A) ZIF-8 and B) APA@ZIF-8 particles before and after the incubation process in PBS pH 3 (a) 0 h, (b) 30 min, (c) 1 h, (d) 2h, (e) 4h, (f) 6h, (g) 12h, (h) 24h, (i) 48h. **(B)** PXRD patterns illustrating the structural evolution of A) ZIF-8 and B) APA@ZIF-8 particles before and after the incubation process in PBS pH 5 (a) 0, h (b) 30 min, (c) 1 h, (d) 2h, (e) 4h, (f) 6h, (g) 12h, (h) 24h, (i) 48h.

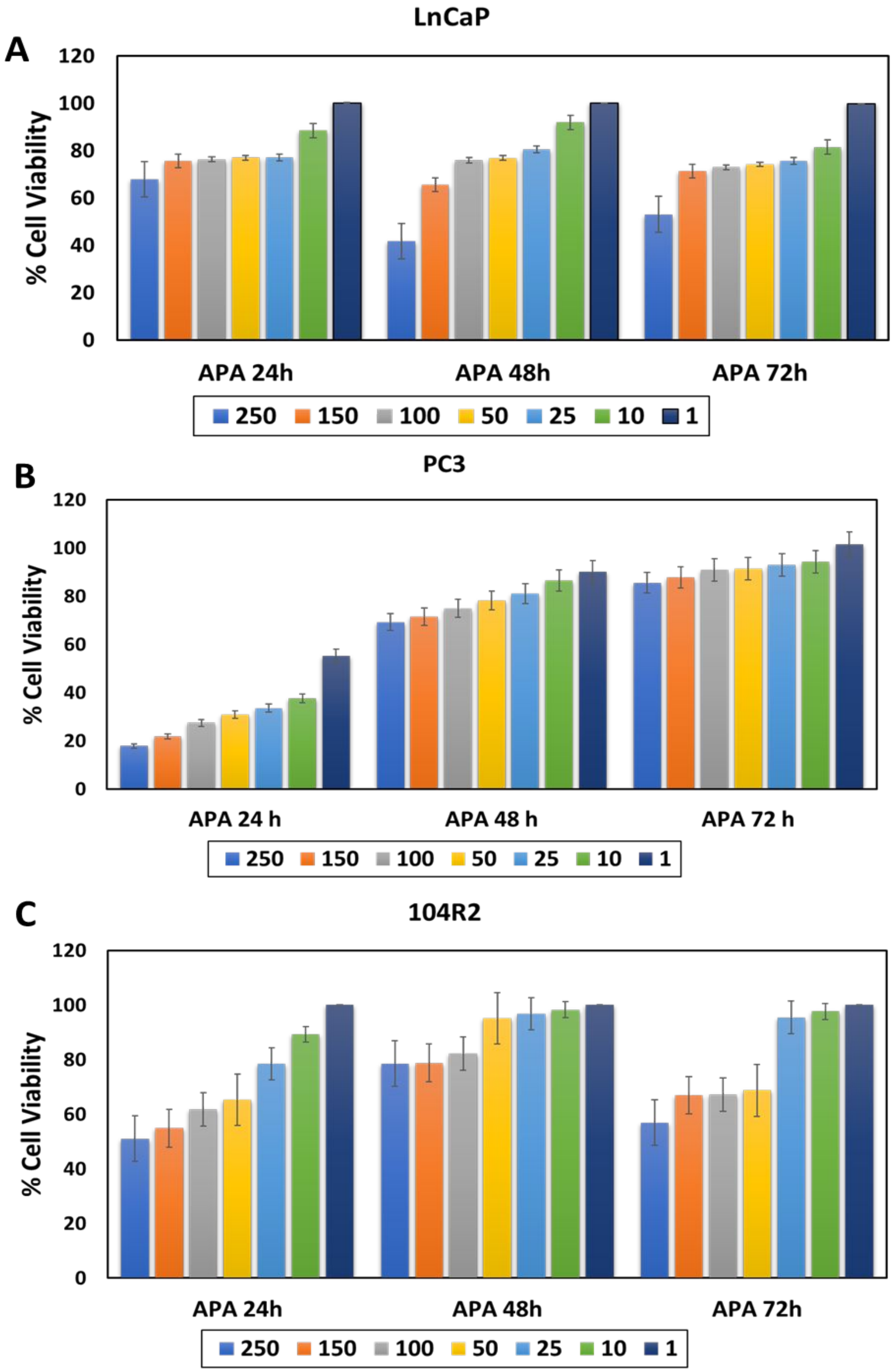

Figure S2. Cytotoxic activity profiles APA in a) LnCaP, b) PC3, and c) 104R2 cell lines at 24, 48, and 72 hours.
